# AI-Guided Multi-Omic Microbiome Modulation Improves Clinical and Inflammatory Outcomes in Refractory IBD: A Real-World Study

**DOI:** 10.3390/ijms27010201

**Published:** 2025-12-24

**Authors:** Raluca Lupusoru, Lavinia Cristina Moleriu, Ruxandra Mare, Ioan Sporea, Alina Popescu, Roxana Sirli, Adrian Goldis, Camelia Nica, Tudor Voicu Moga, Bogdan Miutescu, Iulia Ratiu, Oana Belei, Laura Olariu, Victor Dumitrascu, Radu Dumitru Dragomir

**Affiliations:** 1 Department III, Functional Science, Discipline of Medical Informatics and Biostatistics, “Victor Babes” University of Medicine and Pharmacy, 300041 Timisoara, Romania; raluca.lupusoru@umft.ro; 2 Center for Modeling Biological Systems and Data Analysis, “Victor Babes” University of Medicine and Pharmacy, 300041 Timisoara, Romania; 3 Gastroenterology and Hepatology Clinic, County Emergency Hospital “Pius Brinzeu”, 300723 Timisoara, Romania; isporea@umft.ro (I.S.); popescu.alina@umft.ro (A.P.); sirli.roxana@umft.ro (R.S.); goldis.adrian@umft.ro (A.G.); camelia.foncea@umft.ro (C.N.); moga.tudor@umft.ro (T.V.M.); miutescu.bogdan@umft.ro (B.M.); ratiu.iulia@umft.ro (I.R.); 4 Advanced Regional Research Center in Gastroenterology and Hepatology, “Victor Babes” University of Medicine and Pharmacy, 300041 Timisoara, Romania; 5 Department of Internal Medicine II, Division of Gastroenterology and Hepatology, “Victor Babes” University of Medicine and Pharmacy, 300041 Timisoara, Romania; 6 First Pediatric Clinic, “Victor Babes” University of Medicine and Pharmacy, 300041 Timisoara, Romania; belei.oana@umft.ro (O.B.); olariu.laura@umft.ro (L.O.); 7 Disturbances of Growth and Development on Children Research Center, “Victor Babes” University of Medicine and Pharmacy, 300041 Timisoara, Romania; 8 Department IV, Biochemistry and Pharmacology, Discipline of Pharmacology, “Victor Babes” University of Medicine and Pharmacy, 300041 Timisoara, Romania; dumitrascu.victor@umft.ro; 9 Department of Oncology, “Victor Babes” University of Medicine and Pharmacy, 300041 Timisoara, Romania; radu.dragomir@umft.ro

**Keywords:** inflammatory bowel disease (IBD), multi-omics, gut microbiome modulation, artificial intelligence–guided therapy, immunometabolism, microbiome–immune interactions, personalized medicine, short-chain fatty acid–producing bacteria

## Abstract

Inflammatory bowel disease (IBD) remains difficult to manage in patients who fail multiple therapeutic lines, and growing evidence suggests that alterations in the gut microbiome contribute to persistent symptoms and inflammatory activity. This study evaluated a three-month, AI-guided, multi-omic personalized microbiome modulation program in adults with treatment-refractory IBD. Baseline stool metagenomic sequencing, blood biomarkers, micronutrient panels, and clinical data were integrated through an artificial intelligence platform to generate individualized plans combining dietary adjustments, targeted synbiotics, selective antimicrobials, and micronutrient correction. Clinical outcomes, inflammatory markers, and microbial signatures were reassessed after three months. Across 358 participants, stool frequency decreased substantially, urgency and rectal bleeding resolved in most patients, and over 70% reported a “much improved” overall condition. Inflammatory biomarkers showed marked normalization, with reductions in hs-CRP and fecal calprotectin observed in over 85% of cases. Micronutrient deficiencies, particularly iron and zinc, also improved, and beneficial microbial taxa such as *Faecalibacterium prausnitzii*, *Bifidobacterium longum*, and *Akkermansia muciniphila* increased significantly. These findings suggest that personalized, multi-omic microbiome modulation may support clinically meaningful improvements by targeting microbial, metabolic, and immune imbalances rather than symptoms alone. While encouraging, these results require confirmation in randomized controlled studies.

## 1. Introduction

Inflammatory bowel disease (IBD), which includes ulcerative colitis (UC) and Crohn’s disease (CD), remains a complex, chronic condition characterized by unpredictable relapses, progressive intestinal damage, and significant impairment of quality of life [1]. Over the past decades, its global prevalence has risen steadily, transforming IBD from a regional concern to a worldwide public health issue [2]. Patients often face lifelong therapy, recurrent hospitalizations, and a heavy psychosocial burden, while healthcare systems must absorb the growing economic costs associated with chronic management [3].

Despite the evolution of therapeutic options, from corticosteroids and immunomodulators to biologics and, more recently, small-molecule inhibitors, long-term disease control remains elusive for many [4]. A substantial proportion of patients experience primary non-response to biologics, while others gradually lose efficacy over time [5]. Even when clinical remission is achieved, residual mucosal inflammation or subclinical activity frequently persists [6]. Moreover, the chronic suppression of immune pathways carries well-documented risks, including infections and systemic complications [7]. These realities highlight a critical limitation of the current treatment paradigm: most available therapies act downstream, targeting inflammation rather than its root biological drivers [8].

In recent years, attention has increasingly turned toward the gut microbiome, a dynamic ecosystem of bacteria, fungi, viruses, and archaea, as a central player in IBD pathogenesis [9]. Alterations in microbial diversity and metabolic function, known collectively as dysbiosis, contribute to immune activation, epithelial barrier disruption, and chronic mucosal injury [10]. Gut microbiome dysbiosis contributes to both the initiation and persistence of inflammation in IBD through multiple interconnected mechanisms. Reduced microbial diversity and depletion of beneficial commensals impair the production of short-chain fatty acids, particularly butyrate, which are essential for epithelial energy metabolism, maintenance of tight junction integrity, and anti-inflammatory immune signaling. In parallel, the expansion of pro-inflammatory pathobionts increases luminal antigen load, promotes epithelial adherence and translocation, and amplifies innate and adaptive immune activation. This dysregulated host–microbiome interaction establishes a self-perpetuating cycle of barrier dysfunction, immune stimulation, and ongoing mucosal inflammation, even in the absence of overt clinical disease activity.

Several studies have demonstrated that IBD patients harbor reduced levels of beneficial bacterial species such as *Faecalibacterium prausnitzii* and *Bifidobacterium* spp., alongside an expansion of pro-inflammatory species like Enterobacteriaceae. These alterations are not merely descriptive, but functionally relevant, as the depletion of SCFA-producing commensals such as *Faecalibacterium prausnitzii* has been associated with impaired epithelial barrier integrity, reduced anti-inflammatory signaling, and increased mucosal immune activation. Conversely, the expansion of Enterobacteriaceae has been linked to enhanced oxidative stress, epithelial adherence, and perpetuation of inflammatory cascades in IBD [11,12,13]. While these findings have deepened our understanding of disease mechanisms, translating them into effective, patient-specific therapies remains challenging.

Conventional approaches, such as dietary interventions, probiotics, or fecal microbiota transplantation, have produced variable outcomes, largely due to the extraordinary inter-individual variability in microbial composition and host response. Specifically, these studies demonstrate that standardized dietary patterns, probiotic formulations, or donor-dependent microbiota transfer protocols often fail to account for baseline microbial heterogeneity, functional metabolic differences, and host immune context. As a result, identical interventions may induce beneficial microbial shifts in some individuals while remaining neutral or even exacerbating dysbiosis in others. This variability underscores the limitations of non-personalized microbiome interventions and highlights the need for individualized strategies informed by baseline microbiome and host-specific characteristics [14,15]. The same intervention that benefits one patient may be ineffective or even detrimental to another. As a result, the future of microbiome-centered therapy lies not in standardized regimens, but in personalized modulation guided by precise biological data [16].

Advances in shotgun metagenomic sequencing and multi-omic profiling (including blood metabolomics, cytokine analysis, and micronutrient mapping) now allow a high-resolution view of each patient’s microbial and host landscape [17]. However, integrating and interpreting these large datasets requires advanced computational methods capable of revealing actionable patterns. Artificial intelligence (AI) provides exactly this capability, enabling the fusion of diverse omic layers with clinical and lifestyle data to design tailored interventions [18,19,20].

The present study builds upon this concept by employing an AI-driven, multi-omic personalization framework for microbiome modulation in IBD. Using the NostraBiome platform, baseline stool metagenomics and blood biomarkers were integrated with patient history to generate individualized plans combining dietary adjustments, targeted synbiotics, selective antimicrobials, and micronutrient correction.

The study aimed to quantify clinical improvement, changes in inflammatory activity, and quality-of-life enhancement after three months of intervention. Beyond validating the efficacy of this integrative model, our findings provide early evidence that personalized microbiome modulation (when guided by multi-omic data and AI) may represent a viable and scalable adjunct to conventional IBD management.

## 2. Results

All predefined study objectives were achieved, with coherent improvements observed across clinical, biochemical, and microbiome domains after three months of personalized, AI-guided microbiome modulation. The findings confirm a consistent therapeutic benefit that extends beyond symptom control, reflecting deeper metabolic and immunological balance. No adverse events were recorded throughout the study.

The study included a cohort of adults with moderate to severe treatment-refractory IBD. The cohort represented a balanced population in terms of age, sex, and body mass index, with most patients having a history of exposure to multiple prior therapeutic lines, including biologics and corticosteroids. The baseline demographic and clinical characteristics of the study population are summarized in Table 1.

Following three months of AI-guided, personalized microbiome modulation, patients demonstrated a marked improvement in bowel function and overall clinical status.

Specifically, mean stool frequency decreased from 8.87 ± 2.05 bowel movements per day at baseline to 2.76 ± 1.11 at Month 3, corresponding to a mean reduction of 6.11 bowel movements per day (*p* < 0.001). Urgency and rectal bleeding resolved in 327 of 358 patients (91.3%), indicating near-complete normalization of these key clinical symptoms. Overall, more than 70% of patients rated their clinical condition as “much improved” after three months of intervention. Stool frequency decreased substantially from baseline, accompanied by normalization of stool consistency and the resolution of rectal bleeding and urgency in most participants. The overall clinical response was rated as “Much improved” for the majority of patients, indicating a consistent therapeutic response and rapid symptom stabilization. Detailed clinical outcome measures are presented in Table 2.

**Figure 1:**
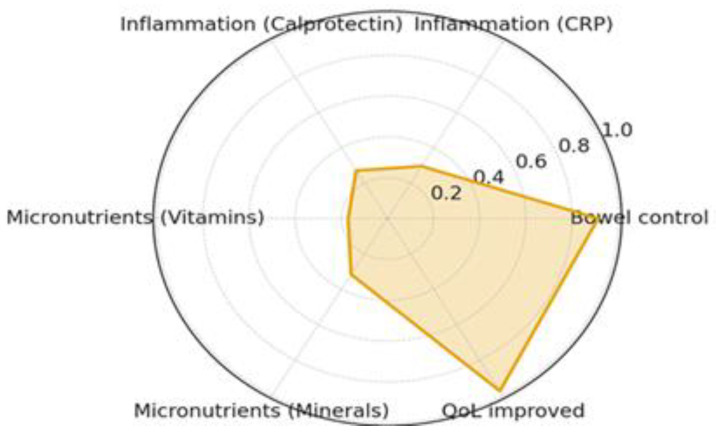
Integrated clinical domains of response following personalized microbiome modulation.

**Figure 2:**
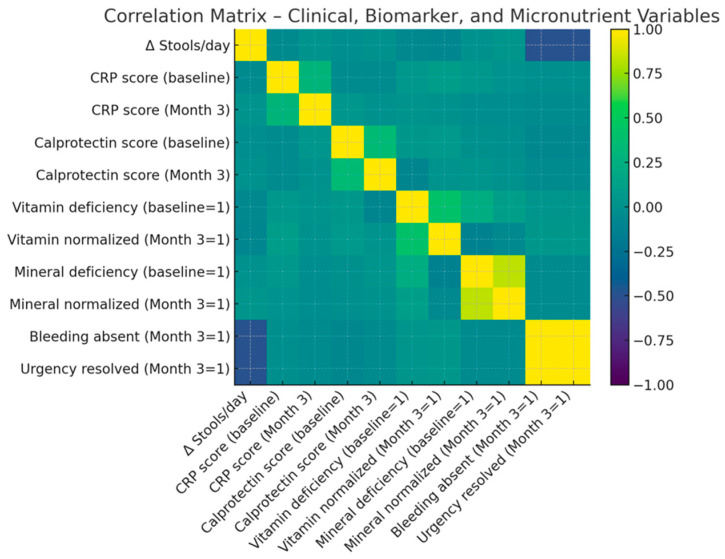
Correlation matrix between clinical, inflammatory, and biochemical parameters at Month 3.

The reduction in inflammatory activity closely matched the clinical improvement observed across the cohort. Both systemic and intestinal biomarkers indicated marked normalization by Month 3, indicating effective suppression of inflammatory burden and restoration of mucosal equilibrium. In parallel, a substantial proportion of patients exhibited correction of baseline micronutrient deficiencies, reflecting improved absorptive and metabolic function. Detailed changes in inflammatory biomarkers and micronutrient deficiencies following the personalized microbiome intervention are summarized in Table 3 and illustrated in Figure 1 and Figure 2.

Figure 1 illustrates the multidimensional improvement observed across major domains after three months of AI-guided, multi-omic microbiome modulation in patients with inflammatory bowel disease. Each axis represents a key therapeutic outcome—bowel control, inflammatory activity (hs-CRP and fecal calprotectin), micronutrient normalization (vitamins and minerals), and quality of life (QoL). Values are normalized between 0 and 1, with larger surface areas indicating greater overall recovery and balanced improvement across domains.

Figure 2 illustrates Pearson correlation coefficients among key clinical variables, inflammatory biomarkers, and micronutrient parameters before and after the 3-month personalized microbiome modulation program. Notable positive correlations (yellow) indicate aligned improvement across domains such as reduced stool frequency, normalization of calprotectin and hs-CRP, and resolution of urgency and bleeding. Negative correlations (purple/blue) reflect inverse relationships between baseline abnormalities and post-intervention recovery. This multidimensional pattern highlights the interconnected nature of inflammatory control, metabolic correction, and clinical remission in treatment-refractory IBD.

**Figure 3:**
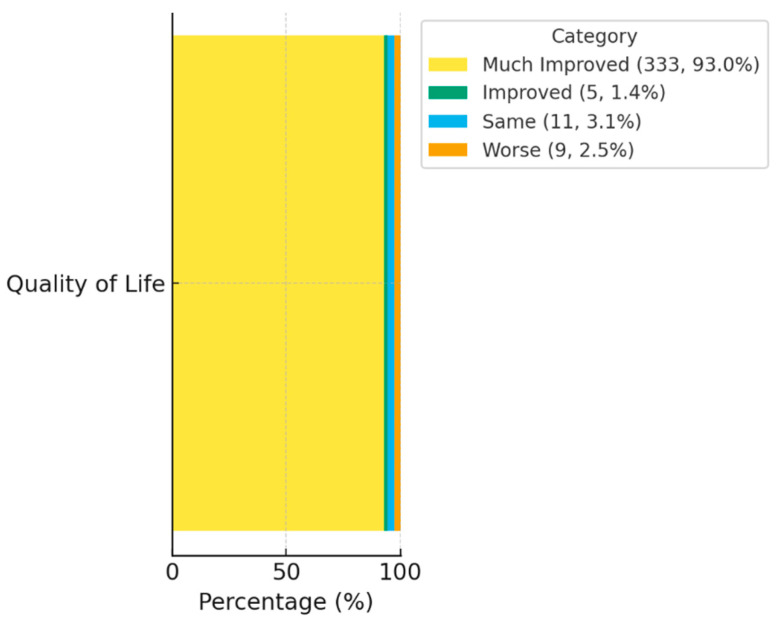
Quality-of-life improvement following AI-guided, multi-omic microbiome modulation.

Figure 3 illustrates the distribution of patient-reported outcomes according to the SIBDQ. After three months of intervention, the majority of participants reported a “Much improved” quality of life, with only a small fraction reporting minimal or no change. These results suggest that biological improvements were paralleled by enhanced perceived well-being and daily functioning.

Table 4 summarizes categorical improvements in the abundance of key commensal bacterial species associated with gut homeostasis and anti-inflammatory activity. Each taxon was evaluated based on ordinal changes (low → normal → high) after three months of AI-guided, multi-omic microbiome modulation. Notable increases were observed in *Bifidobacterium longum*, *Faecalibacterium prausnitzii*, and *Roseburia intestinalis*, all known producers of short-chain fatty acids that support mucosal healing and immune regulation.

**Figure 4:**
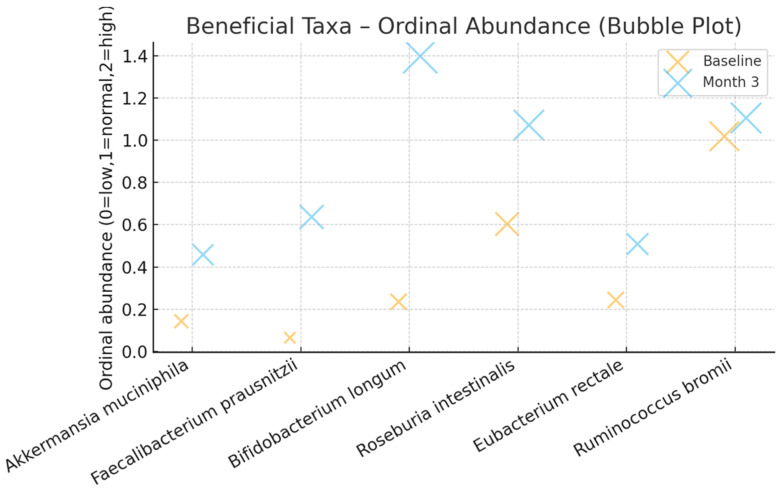
Changes in beneficial microbial taxa following personalized microbiome modulation.

Figure 4 displays the ordinal abundance of key beneficial taxa at baseline and after three months of AI-guided, multi-omic microbiome modulation. Each point represents the mean ordinal abundance score (0 = low, 1 = normal, 2 = high), with bubble size proportional to mean relative abundance. All reported taxonomic changes were statistically significant after false discovery rate (FDR) correction (q < 0.05), confirming the robustness of the observed microbial shifts. Increases in the abundance of short-chain fatty acid-producing species such as *Faecalibacterium prausnitzii*, *Bifidobacterium longum*, *Roseburia intestinalis*, and *Akkermansia muciniphila* indicate microbiome repair and metabolic recovery, which is congruent with clinical and inflammatory improvements.

## 3. Discussion

Our findings suggest that personalized, AI-guided microbiome modulation is associated with clinically meaningful and biologically coherent improvement in refractory IBD [21]. Taken together, symptom resolution, biomarker normalization, and beneficial taxonomic shifts support a mechanism consistent with upstream restoration of intestinal ecosystem homeostasis across microbial, immune, and metabolic axes [22,23].

The magnitude of improvement observed in our cohort is notable for a population that had failed multiple prior therapies. Pivotal infliximab trials in ulcerative colitis (ACT 1 and ACT 2) reported clinical remission rates of 34–39% at week 8 [24], while adalimumab induction studies (ULTRA 2) demonstrated remission in 16–21% of patients [25]. In contrast, more than 70% of participants in our cohort described themselves as “much improved” after three months of AI-guided microbiome modulation. Importantly, these outcomes were achieved without systemic immunosuppression, underscoring the potential of microbiome-based therapies as a safe, non-pharmacologic adjunct to standard care.

The need for alternative IBD therapies arises from key limitations of current immune-suppressive treatments, including primary and secondary non-response, incomplete mucosal healing, and long-term safety concerns. Moreover, these approaches do not address upstream contributors such as dysbiosis, epithelial barrier impairment, and immunometabolism dysfunction, supporting the exploration of microbiome-targeted strategies.

Previous research on dietary or probiotic interventions in IBD has yielded modest or inconsistent effects [26,27,28,29]. Randomized trials of standard probiotics, such as *E. coli*, have demonstrated benefit primarily in mild ulcerative colitis, with little impact on CD or refractory phenotypes [30,31]. In contrast, our data show comparable improvements across both UC and Crohn’s subgroups, supporting the concept that individualized modulation, grounded in metagenomic and biochemical data, can overcome the inter-individual variability that limits generic microbiome therapies.

The concordant improvement in clinical and biochemical markers is consistent with mechanisms involving immune recalibration and epithelial repair [32,33,34]. The expansion of SCFA-producing commensals (e.g., *Faecalibacterium prausnitzii*, *Bifidobacterium longum*, *Roseburia intestinalis*) together with increased *Akkermansia muciniphila* supports improved barrier function and immune regulation, consistent with prior links to mucosal healing and reduced relapse risk [35,36,37,38].

Inflammatory normalization (particularly the reduction in hs-CRP and fecal calprotectin in over 85% of patients) provides biochemical validation of this mechanism [39]. Prior studies using fecal microbiota transplantation (FMT) have reported more modest reductions in calprotectin, often transient and dependent on donor compatibility [40]. The reproducible biomarker response in our study suggests personalized modulation may achieve more consistent biomarker improvement than empirical approaches [41].

The parallel improvement in inflammatory biomarkers and clinical symptoms observed in our cohort is consistent with the treat-to-target framework in IBD, where biomarker normalization is expected to accompany (and often precede) sustained symptomatic control and mucosal recovery. Fecal calprotectin is a sensitive marker of intestinal neutrophilic inflammation and has been repeatedly associated with symptom burden and patient-reported outcomes, including stool frequency, urgency, and rectal bleeding; thus, declining calprotectin levels provide objective support for clinically meaningful improvement rather than symptomatic fluctuation alone. Similarly, reductions in systemic inflammation reflected by hs-CRP correlate with decreased inflammatory activity and lower risk of ongoing disease burden in responsive patients. In our study, the high normalization rates of fecal calprotectin and hs-CRP occurred alongside resolution of urgency and rectal bleeding and marked reductions in stool frequency, reinforcing the biological coherence between symptomatic recovery and inflammatory control described in prior longitudinal analyses and guideline-driven treat-to-target approaches.

Beyond mucosal healing, metabolic recovery appears to have played a role [18]. Normalization of vitamin and mineral deficiencies implies improved nutrient absorption and reduced inflammatory consumption, both likely consequences of epithelial repair [42]. The interplay between microbial metabolism and host micronutrient status is well documented: SCFA-producing taxa promote mineral bioavailability, while Bifidobacterium species contribute to endogenous synthesis of B vitamins [43]. The parallel normalization of these parameters in our study underscores the integrated nature of the microbiome–metabolome axis in sustaining mucosal and systemic health [44].

A unique strength of this study lies in the multidimensional coherence of its outcomes [45]. The radar and correlation analyses revealed that improvements in stool control, inflammatory burden, micronutrient balance, and quality of life occurred in parallel, suggesting that the intervention achieved systemic rather than isolated effects [43]. This integrated pattern differentiates microbiome modulation from conventional immunosuppressive therapies, which often yield biochemical remission without full symptomatic or metabolic recovery [46]. Notably, objective inflammatory improvements aligned with patient-reported quality-of-life gains, supporting clinically relevant recovery across domains [47,48,49].

The therapeutic outcomes reported here compare favorably with other microbiome-targeted approaches in IBD. FMT, though conceptually similar, is limited by donor variability, engraftment instability, and procedural invasiveness [50]. Dietary exclusion protocols and fiber-enriched regimens show modest improvements but are difficult to sustain and lack precision [32]. In contrast, the AI-curated, multi-omic strategy employed in this study combines the mechanistic depth of omic profiling with the adaptability of individualized design [18]. By continuously integrating metagenomic, biochemical, and symptomatic data, this approach mimics a “living therapy” that adjusts dynamically to patient response, an advancement beyond static nutritional or probiotic prescriptions.

Our findings align with recent data from integrative nutrition and precision medicine studies that emphasize the need to personalize microbiome interventions according to microbial and metabolic signatures [18]. For instance, Zeevi et al. (Cell, 2015) [18] demonstrated that individual glycemic and inflammatory responses to identical foods are highly variable and microbiome dependent [42]. The significant heterogeneity observed in IBD treatment outcomes likely follows a similar logic: what exacerbates dysbiosis in one patient may restore balance in another [22]. The use of artificial intelligence to interpret these complex interactions and guide adaptive interventions represents a paradigm shift in the management of chronic inflammatory diseases [51].

These results highlight the potential of precision microbiome medicine as a safe and effective complement to standard IBD therapy. The absence of adverse events and the high adherence rate are particularly encouraging in a cohort that had exhausted multiple pharmacologic options [52]. The improvement across both UC and CD subtypes indicates that this strategy targets shared pathophysiologic pathways, namely epithelial integrity, microbial diversity, and metabolic-immune crosstalk, rather than disease-specific immune mediators [53,54].

Importantly, this integrative model redefines how therapeutic success in IBD can be conceptualized. Such an approach aligns with a growing body of literature proposing that the microbiome is not merely a biomarker of disease activity, but an active driver of remission or relapse. By leveraging multi-omics data and AI to personalize intervention, the present study provides real-world evidence that targeted ecological restoration may contribute to clinical and biochemical improvement in refractory IBD [51].

From a broader physiopathological standpoint, these findings support the hypothesis that IBD is fundamentally a disorder of disrupted host–microbiome symbiosis [55]. The chronic cycle of dysbiosis, epithelial damage, immune activation, and further dysbiosis forms a self-sustaining loop that perpetuates inflammation [56,57]. The results presented here suggest that personalized microbiome modulation can interrupt this loop at multiple points: by reintroducing beneficial species, enhancing barrier function, reducing luminal antigen load, and restoring metabolic harmony [58,59,60]. Together, these observations support precision microbiome modulation as a plausible adjunct strategy for refractory IBD that warrants confirmation in randomized controlled trials with endoscopic endpoints and longer follow-up.

### Strengths, Limitations, and Future Directions

The present study represents one of the largest real-world analyses to date evaluating an AI-guided, multi-omic microbiome modulation program in patients with inflammatory bowel disease. Its major strengths include the comprehensive integration of metagenomic, biochemical, and clinical data within a precision framework, and the consistent improvement observed across independent domains, clinical, inflammatory, nutritional, and microbial, which lends internal validity and biological plausibility to the findings. The use of high-resolution multi-omic profiling and continuous AI-driven adaptation allowed for a meaningful level of personalization in a refractory IBD population. Moreover, the absence of serious adverse events and the high adherence rate reinforce the safety and feasibility of this precision-medicine model in a real-world context.

However, several important limitations must be acknowledged to ensure transparent interpretation of the results.

First, this was a single-arm, open-label study without a randomized control group or placebo comparator. As such, causal inference cannot be established, and improvements observed over the 3-month period may partially reflect non-specific factors, including regression to the mean, increased patient engagement due to digital monitoring, or expectation effects. Although the magnitude and coherence of clinical, biochemical, and microbial changes notably suggest a true biological signal, controlled studies are required to confirm this.

Second, the absence of blinding introduces potential performance and reporting biases, as participants were aware of receiving an active intervention. The use of digital self-reporting tools, while valuable for longitudinal tracking, may also be subject to recall bias and social desirability effects. Additionally, the cohort likely represents a self-selected, digitally literate population motivated to pursue non-pharmacologic therapies, which could limit generalizability to the broader IBD population.

Third, although the AI platform (NostraBiome) integrated diverse omic and clinical layers, the algorithm’s internal parameters, feature weights, and decision thresholds remain proprietary and were not independently audited in this study. This lack of algorithmic transparency and external validation limits reproducibility and prevents full mechanistic understanding of how individual recommendations were derived.

Fourth, while inflammatory and biochemical biomarkers were measured objectively, endoscopic and histologic endpoints were not included, precluding direct assessment of mucosal healing. The 3-month follow-up window, although adequate for initial validation, does not provide insight into long-term durability, relapse prevention, or the sustainability of microbial reconfiguration. Furthermore, certain microbial outcomes were reported categorically (improved vs. not improved) rather than quantitatively, potentially underestimating subtle ecological shifts.

Finally, confounding influences such as minor dietary or lifestyle changes outside the prescribed plan, unreported supplement use, or environmental exposures cannot be completely excluded, despite close digital monitoring.

Building upon these findings, the next logical step is the initiation of a randomized, double-blind, controlled clinical trial to validate the efficacy and mechanistic underpinnings of AI-guided, multi-omic microbiome modulation in IBD. Such a study should compare this personalized approach with both standard-of-care pharmacologic regimens and non-personalized microbiome interventions (e.g., fixed probiotic or dietary protocols). Beyond clinical outcomes, the trial will aim to evaluate objective endoscopic and histologic healing, immune and metabolic pathway modulation, and durability of remission over an extended 12–24-month follow-up period.

This future validation effort will provide the causal evidence necessary to establish personalized microbiome modulation as a scientifically grounded and clinically actionable component of precision IBD management.

Future research should build upon these findings through rigorously designed randomized controlled trials that integrate parallel mechanistic investigations alongside clinical endpoints. Such studies should aim to delineate causal pathways linking microbiome modulation to immune, metabolic, and epithelial responses, rather than relying solely on associative clinical outcomes. The incorporation of longitudinal multi-omic sampling, including metagenomics, metabolomics, transcriptomics, and host immune profiling, will be essential to identify predictive, prognostic, and potentially targetable biomarkers of response to personalized microbiome interventions.

Importantly, future studies should move beyond species-level taxonomic resolution and investigate microbial changes at the strain level. Whole-genome sequencing and functional annotation of beneficial and potentially pro-inflammatory strains will be critical, as distinct strains within the same species may exert markedly different biological effects on host immunity, epithelial integrity, and metabolic signaling. Strain-level characterization will enable more precise identification of functionally relevant microbes and may facilitate the development of targeted, next-generation microbiome therapeutics tailored to individual host–microbiome interactions.

## 4. Materials and Methods

### 4.1. Study Design and Study Population

This was a real-world, non-randomized exploratory study intended to generate preliminary clinical and biological insights, rather than to establish causality.

This work is conducted as a prospective, single-arm, open-label validation study aimed at assessing the clinical and biochemical effects of an AI-guided, multi-omic personalized microbiome modulation program in patients with IBD.

The study consisted of a 3-month active intervention period, followed by a 1-month safety follow-up.

Detailed inclusion (Table 5) and exclusion criteria (Table 6) were designed to ensure a clinically homogeneous, treatment-refractory IBD cohort, free from confounding inflammatory or infectious processes. They also guarantee methodological consistency and data integrity across multi-omic and digital datasets, while preserving participant safety and ethical compliance.

### 4.2. Baseline Multi-Omic Assessment

At baseline, each participant underwent an integrated biological and digital assessment consisting of:

- Stool metagenomic sequencing using Illumina NovaSeq technology, with comprehensive taxonomic, functional, and resistome annotation.
- Blood biomarker profiling, including high-sensitivity C-reactive protein (hs-CRP) and a targeted micronutrient panel (vitamin B12, vitamin D, iron, zinc, calcium).
- Digital lifestyle and symptom assessment, capturing medical history, dietary habits, medication exposure, and prior flare patterns through a structured online questionnaire.

### 4.3. AI-Driven Personalized Intervention

The personalized intervention was guided by the NostraBiome Microbiome Intelligence Technology Platform, an artificial intelligence–based, multi-omic analytics system developed for integrative microbiome interpretation. The platform combines shotgun metagenomic sequencing, metabolomic and transcriptomic profiling, and clinical biomarker data to generate individualized therapeutic recommendations for each patient.

The NostraBiome algorithm employs supervised machine-learning models (gradient-boosted decision trees with ensemble feature selection) trained on a large-scale, curated knowledge base of over one million scientific sources, clinical publications, and validated medical inputs, previously correlated with microbiome-related clinical outcomes. This model enables the identification of inflammatory microbiome signatures, such as the overrepresentation of pathobionts (*Bacteroides fragilis*, *Clostridium difficile*, *Escherichia coli*) and depletion of beneficial taxa (*Faecalibacterium prausnitzii*, *Akkermansia muciniphila*).

### 4.4. Data Collection and Monitoring

Participants recorded daily bowel habits through a secure digital diary integrated within a mobile application. Entries included stool frequency, Bristol Stool Form Scale classification, presence of visible blood, and urgency.

At the 3-month visit, stool and blood samples were recollected for re-analysis, and all patient-reported outcomes and quality-of-life scores were reassessed. Adverse events were actively monitored throughout the study period.

The final intervention plan typically included:

- Precision dietary guidance, adjusting macronutrient ratios and excluding patient-specific pro-inflammatory foods.
- Tailored symbiotic formulations, consisting of strain-level probiotics and prebiotic fibers selected to enhance beneficial microbial functions.
- Targeted antimicrobials or phage therapy applied only when pathogenic signatures exceeded defined thresholds.
- Micronutrient correction for documented deficiencies identified through baseline multi-omic assessment.

### 4.5. Outcome Measures

The primary endpoint was the change from baseline to month 3 in a composite bowel-control index, which combined stool frequency, stool consistency, and the presence or absence of visible blood.

Secondary endpoints focused on broader markers of clinical and physiological improvement. Specifically, the study evaluated dynamic changes in inflammatory biomarkers, including high-sensitivity C-reactive protein (hs-CRP) and fecal calprotectin, as objective measures of inflammatory burden. Additionally, we assessed the normalization of micronutrient deficiencies identified at baseline, reflecting improved metabolic and absorptive balance. Finally, patient-centered outcomes were captured through the Short Inflammatory Bowel Disease Questionnaire (SIBDQ) and through changes in gastrointestinal symptom clusters, such as bloating, abdominal pain, and excessive gas, providing an integrated view of both biological and quality-of-life improvements during the intervention period.

Exploratory objectives involved integrating multi-omic datasets through machine-learning models to identify molecular predictors of response and mechanistic microbial signatures.

### 4.6. Statistical Analysis

Statistical analyses were performed using GraphPad Prism version 10.0 (GraphPad Software, San Diego, CA, USA) and MedCalc Statistical Software version 22.015 (MedCalc Software Ltd., Ostend, Belgium).

Continuous variables were summarized as mean ± standard deviation (SD) for normally distributed data or as median and interquartile range (IQR) for non-normally distributed variables.

The Shapiro–Wilk test was used to assess normality. For paired comparisons between baseline and 3-month follow-up, paired *t*-tests were applied to normally distributed variables, while Wilcoxon signed-rank tests were used for non-parametric data. Categorical variables were analyzed using the Chi-square or Fisher’s exact test, as appropriate. Correlations between clinical, biochemical, and micronutrient parameters were assessed using Pearson or Spearman correlation coefficients, and correlation matrices were visualized in GraphPad Prism. Changes in inflammatory and biochemical markers were additionally evaluated using repeated-measures ANOVA and linear regression models in MedCalc to account for intra-individual variability.

Differential microbiome abundance and community structure analyses (PERMANOVA, ALDEx2) were performed separately in R version 4.3.1 using specialized packages (vegan, ALDEx2).

All statistical tests were two-tailed, and a *p*-value < 0.05 (or FDR-adjusted q < 0.05 for multi-omic analyses) was considered statistically significant.

### 4.7. Ethical Considerations

The study is conducted in accordance with the principles of the Declaration of Helsinki and was approved by the Ethics Committee of the “Pius Brînzeu” County Emergency Clinical Hospital Timișoara (Approval No. 43/17 December 2024). All participants provided informed consent prior to inclusion. Safety monitoring is conducted by an independent physician on a monthly basis, and all patient data were fully anonymized and stored using AES-256-encrypted cloud infrastructure.

## 5. Conclusions

This study indicates that AI-guided, multi-omic microbiome modulation is associated with consistent and clinically meaningful improvement in patients with treatment-refractory inflammatory bowel disease. Over a three-month intervention period, patients experienced substantial normalization of bowel function, marked reductions in inflammatory biomarkers, and improvement in micronutrient profiles, accompanied by enhanced quality of life and restoration of beneficial microbial taxa.

The alignment between symptomatic recovery, biochemical normalization, and microbial restoration suggests that this personalized intervention acts through integrated mechanisms of epithelial repair, immune regulation, and metabolic rebalancing rather than simple symptom control. The parallel improvement across UC and CD subgroups further supports the robustness and generalizability of the approach.

These findings provide real-world evidence that precision microbiome modulation can achieve measurable clinical, biochemical, and microbiological remission in refractory IBD without the use of systemic immunosuppression. By restoring host–microbiome equilibrium, this strategy establishes a foundation for a new, data-driven therapeutic paradigm that complements and potentially enhances conventional IBD management.

The intervention led to significant increases in key beneficial taxa, including *Faecalibacterium prausnitzii*, *Bifidobacterium longum*, *Akkermansia muciniphila*, and *Roseburia intestinalis*, which are central to IBD pathophysiology through their roles in epithelial barrier maintenance, immune regulation, and short-chain fatty acid production. These microbiome shifts provide mechanistic support for the observed clinical and inflammatory improvements.

## Figures and Tables

**Table 1:** Baseline characteristics and therapy category of the study population.

Characteristic	Value
N (IBD total)	358
Ulcerative Colitis **	215 (60.0%)
Crohn’s Disease **	143 (40.0%)
Age (years) *	42.7 ± 15.3
BMI (kg/m^2^) *	24.5 ± 6.2
Male **	187 (52.2%)
Female **	171 (47.8%)
Therapy category	
Biologics **	242 (67.6%)
Steroids **	81 (22.6%)
Immunomodulators **	12 (3.4%)
Antibiotic **	12 (3.4%)
Nutrition **	9 (2.5%)
5-ASA **	2 (0.5%)

Data are presented as mean ± SD * or n (%) **. Abbreviation: 5-ASA = 5-aminosalicylic acid therapy categories indicate treatments received prior to enrollment.

**Table 2:** Clinical Response and symptom improvement at 3 months.

Measure	Value
Stool frequency (baseline)	8.87 ± 2.05
Stool frequency (Month 3)	2.76 ± 1.11
Change (Month 3-baseline)	−6.11 (*p* < 0.001)
Blood in stool (Absent, n/N)	327/358
Urgency resolved (n/N)	327/358
Overall response: Much Improved (n, %)	256 (71.5%)

**Table 3:** Normalization rates of inflammatory and biochemical parameters at Month 3.

Biomarker	Abnormal at Baseline (n)	Normalized at Month 3 (n)	Normalization Rate (%)
CRP (hs-CRP)	221	201	91.0
Fecal Calprotectin	224	196	87.5
Vitamins (B/D)	193	61	31.6
Minerals (Iron/Zinc/etc.)	143	113	79.0

Normalization rates of inflammatory and biochemical parameters after three months of personalized microbiome modulation. All improvements were statistically significant (*p* < 0.001).

**Table 4:** Improvement in beneficial microbial taxa following personalized microbiome modulation.

Taxon	N with Data	Improved (n)	Improved (%)
*Akkermansia muciniphila*	358	77	21.5
*Faecalibacterium prausnitzii*	358	139	38.8
*Bifidobacterium longum*	358	276	77.1
*Roseburia intestinalis*	358	109	30.4
*Eubacterium rectale*	358	67	18.7
*Ruminococcus bromii*	358	20	5.6

Data represent the number and percentage of patients showing categorical improvement in the relative abundance of key beneficial taxa after three months of personalized microbiome modulation. All changes were statistically significant (*p* < 0.001).

**Table 5:** Inclusion criteria.

Confirmed diagnosis	Documented diagnosis of UC or CD established by endoscopic, histologic, or radiologic evidence consistent with current international guidelines (ECCO/AGA) [61,62].
	Disease duration of at least 24 months prior to baseline sampling, ensuring a chronic and stable diagnostic classification.
Disease activity and treatment history	History of inadequate response, secondary loss of response, or intolerance to at least three previous therapeutic regimens, which may include: Corticosteroids (systemic or topical). Immunomodulators (azathioprine, 6-mercaptopurine, methotrexate). Biologic agents (anti-TNF, anti-integrin, or anti-IL agents). Small-molecule inhibitors (e.g., JAK inhibitors). 5-ASA derivatives, if used appropriately.
	Patients may continue their stable maintenance therapy (if tolerated) during the study, provided no dose adjustments occurred in the 4 weeks preceding enrollment.
Clinical stability	Absence of acute IBD flare requiring hospitalization, intravenous corticosteroids, or biologic induction within 4 weeks prior to study entry.
	No significant changes in concomitant medications, diet, or lifestyle in the month prior to baseline sampling.
Age and consent	Adults aged 18 to 65 years at the time of informed consent.
	Ability and willingness to provide written informed consent and to comply with all digital monitoring, sample collection, and follow-up procedures.
Technical and logistical feasibility	Access to a compatible smartphone or device required for app-based data collection (daily eDiary).
	Agreement to provide serial stool and blood samples at baseline and 3-month follow-up.

**Table 6:** Exclusion criteria.

Recent antimicrobial or probiotic use	Use of systemic or topical antibiotics (oral, intravenous, or intramuscular) within 30 days before baseline sample collection.
	Use of antifungal agents, antiparasitic drugs, or non-standard antimicrobial supplements within the same period.
	Use of non-study probiotics or prebiotics initiated within 4 weeks prior to baseline.
Acute or severe medical conditions	Active gastrointestinal infections (e.g., *Clostridium difficile*, *Salmonella*, *Campylobacter*, *Giardia lamblia*)
	Known intestinal obstruction, perforation, or intra-abdominal abscess.
	Active gastrointestinal bleeding requiring medical intervention.
	Known colorectal carcinoma, dysplasia, or high-grade intraepithelial neoplasia.
Systemic or autoimmune disorders	Concurrent autoimmune diseases (e.g., lupus erythematosus, rheumatoid arthritis, multiple sclerosis) that may confound immunologic or inflammatory endpoints.
	Severe hepatic impairment (AST/ALT > 3× upper limit of normal) [63], renal failure (eGFR < 45 mL/min/1.73 m^2^) [64], or uncontrolled endocrine/metabolic disorders.
Medication and immunosuppression	Current or recent use (within 3 months) of cytotoxic chemotherapy, immunosuppressive agents unrelated to IBD management, or systemic corticosteroids > 20 mg/day of prednisone equivalent.
	Participation in another interventional clinical trial within 12 weeks prior to enrolment.
Pregnancy and reproductive health	Pregnant or breastfeeding women, or those planning pregnancy during the study period.
	Women of childbearing potential not willing to use adequate contraception during the study (barrier, hormonal, or intrauterine methods).
Allergies and contraindications	Known hypersensitivity or allergy to any potential study component (nutritional supplements, probiotics, antimicrobial agents, or excipients).
Psychiatric or cognitive limitations	Cognitive impairment, psychiatric illness, or substance abuse that may interfere with the ability to consent or adhere to the study protocol.
Compliance and data integrity	Failure to complete baseline data entry, or anticipated inability to adhere to digital monitoring and follow-up visits.
	Any condition deemed by the investigator to compromise patient safety or data reliability.

## Data Availability

The original contributions presented in this study are included in the article. Further inquiries can be directed to the corresponding authors.
